# Arsenic Release from Soil Induced by Microorganisms and Environmental Factors

**DOI:** 10.3390/ijerph19084512

**Published:** 2022-04-08

**Authors:** Yitong Yin, Ximing Luo, Xiangyu Guan, Jiawei Zhao, Yuan Tan, Xiaonan Shi, Mingtao Luo, Xiangcai Han

**Affiliations:** 1 School of Ocean Sciences, China University of Geosciences (Beijing), Beijing 100083, China; kaqichuan@163.com (Y.Y.); guanxy@cugb.edu.cn (X.G.); 2011200021@cugb.edu.cn (J.Z.); tanyuan0228@163.com (Y.T.); 2005200052@cugb.edu.cn (X.S.); luomingtao1996@163.com (M.L.); 2001200152@cugb.edu.cn (X.H.); 2 Beijing Key Laboratory of Water Resources and Environmental Engineering, China University of Geosciences (Beijing), Beijing 100083, China; 3 Yantai Coastal Zone China Geological Survey, Yantai 264000, China

**Keywords:** low-molecular-weight organic acid salts, phosphate, arsenic-contaminated soil, microorganisms, nano zero-valent iron (nZVI)

## Abstract

In rhizospheric soil, arsenic can be activated by both biological and abiotic reactions with plant exudates or phosphates, but little is known about the relative contributions of these two pathways. The effects of microorganisms, low-molecular-weight organic acid salts (LMWOASs), and phosphates on the migration of As in unrestored and nano zero-valent iron (nZVI)-restored soil were studied in batch experiments. The results show that As released by microbial action accounted for 17.73%, 7.04%, 92.40%, 92.55%, and 96.68% of the total As released in unrestored soil with citrate, phytate, malate, lactate, and acetate, respectively. It was only suppressed in unrestored soil with oxalate. In restored soil, As was still released in the presence of oxalate, citrate, and phytate, but the magnitude of As release was inhibited by microorganisms. The application of excess nZVI can completely inhibited As release processes induced by phosphate in the presence of microorganisms. Microbial iron reduction is a possible mechanism of arsenic release induced by microorganisms. Microorganisms and most environmental factors promoted As release in unrestored soil, but the phenomenon was suppressed in restored soil. This study helps to provide an effective strategy for reducing the secondary release of As from soils due to replanting after restoration.

## 1. Introduction

Arsenic (As) is one of the most harmful and widespread pollutants in the natural environment, and As-contaminated soils are widespread globally [1]. Hence, the remediation of soils contaminated with arsenic has become a focus of global concern. Recently, Fe-based materials, biochar, and composites have been studied as amendments for stabilizing As in soil [2]. However, further studies have indicated that, among composite materials, Fe-based materials play a significant role in stabilizing As [3,4,5,6,7]. Among the Fe-based materials used for remediation in recent years, nano zero-valent iron (nZVI) has received increasing attention due to its large specific surface area and high reactivity [8].

NZVI is used to immobilize As by promoting the transformation of more mobile As fractions into less mobile fractions. Hou et al. (2020) found that the proportion of amorphous hydrous oxide–bound As and residual As was increased after using a sponge iron filter containing large amounts of zero-valent iron to restore soil [9]. In addition, Li et al. (2020) found that the percentage of the acid-soluble As decreased, while the reducible As increased by 25.4%. Zeolite-supported nZVI has been used to immobilize As in alkaline soils [10]. These studies on the transformation of soil As fractions indicate that soil restoration efforts can be effective, because the application of nZVI enhances the conversion of soluble As to the insoluble fraction. However, several studies have found that the amorphous hydrous oxide–bound As and the reducible As fraction are potentially bioavailable [11]. Additionally, An et al. (2019) suggested that chemical analysis alone is insufficient to assess the ecotoxicological responses of As in soil. Therefore, further assessment of the biological responses of restored soil is needed to test the stability of the in situ immobilization of As under actual replanting conditions [12].

The biogeochemical cycle of As involves several physical and chemical processes (precipitation/solubilization, adsorption/desorption, and redox processes), as well as biological processes, especially those involving microorganism reactions [13]. Many studies have verified that microorganisms are a key mediator of the biogeochemical release and activation of As [14,15,16]. Several of the potential mechanisms of microbial involvement in As release have been verified and summarized, including As desorption from adsorption sites and As release by the reductive dissolution of iron minerals [17,18]. In recent years, it has been accepted that the development of anoxic conditions in soils leads to increased As mobility, mainly through direct As(V) reduction to As(III) and reductive dissolution of Fe(III) minerals [19]. This poses a challenge to practical applications in contaminated sites, because with either direct plant cultivation or replanting after remediation, processes such as rhizospheric interaction and fertilization may cause changes in the soil environment. These changes may also affect the re-release of As via adsorption/desorption processes or the dissolution of iron minerals.

Plants exude large amounts of photosynthesis-derived carbon (11–40%) via root exudates [20]. Among them, low-molecular-weight organic acids (LMWOAs), as one of the main exudates, are usually in a dissociated mildly acidic state [21]. Acetic acid, oxalic acid, malic acid, and citric acid are typical LMWOAs present in plant root exudates [22]. However, unlike typical plants, phytate (inositol hexaphosphate) has been detected in the root exudates of ferns such as *Pteris vittata* L. (Chinese Brake fern) [23]. As well as being active components of root exudates that can mobilize nutrients such as Fe and P, LMWOAs are also important sources of soil organic carbon [24]. Compared to complex organic matter (e.g., humic acid), microorganisms often utilize LMWOAs as available labile carbon sources [21]. The diversity of microbial community structures and the dynamics of phylogenetic composition can also be regulated by LMWOAs [25]. In addition to being utilized as organic carbon by microorganisms, LMWOAs can impact the soil environment via their functional groups. LMWOAs are often used as soil leaching reagents for soil washing because of their functional groups and organic ligands [2,26,27]. Several experiments have demonstrated the ability of LMWOAs to extract Fe-bound As and residual As from soil [28]. Therefore, replanting on stabilized soils is likely to induce the release of As from the rhizosphere environment.

Phosphorus (P) fertilizers are usually applied to promote plant growth. Phosphate (PO_4_^3−^), the main component of phosphate fertilizers, has multiple influences on As bioavailability. Phosphate can significantly suppress the adsorption of As to iron (hydr)oxides and to soils because of the structural similarity between phosphate (PO_4_^3−^) and arsenate (AsO_4_^3−^). Ji et al. (2019) and Deng et al. (2020) described in detail the effects of phosphate on As behavior in paddy soils [29,30]. Phosphorus is also an essential nutrient for both crops and microorganisms; however, both arsenate and phosphate can be taken up by bacteria via the same phosphate transporters, such as Pst and Pit [31]. Wang et al. (2020) demonstrated the occurrence of phosphate-stimulated As(V) reduction via faster bacterial reproduction and accelerated As desorption/sorption mediated by Bacillus XZM and suggested that phosphate may regulate the biogeochemical behavior of As [32].

This poses a challenge to practical applications in contaminated sites, because with either direct plant cultivation or replanting after remediation, processes such as rhizospheric interaction and fertilization may cause changes in the soil environment. The presence of LMWOAs and phosphate influences As re-release in the soil via both physicochemical and microbial processes. There are many studies of the effects of phosphate on As behavior with the participation of microorganisms in soils. More consideration has been given to the physicochemical effects of LMWOAs on As migration in soils [33,34,35,36,37,38]. However, there has been comparatively little consideration of the role of microorganisms in the action of LMWOAs [39,40]. Many studies have demonstrated that As release is closely related to Fe during the reaction process between As and LMWOAs or phosphate [29,30,35,36,37]. Therefore, we need to monitor the changes to Fe throughout the whole reaction process, including Fe(II) and Fe(III). In addition, the effects of these influencing factors on the re-release of As in soils remediated with nZVI have not been investigated in detail.

In this study, we evaluated the effectiveness of excess nZVI for the remediation of As-contaminated soils. The study area comprises a transition zone between agricultural soils and river sediments and has not previously been cultivated. The objectives of the study were as follows: (1) to determine the influence of low-molecular-weight organic acid salts (LMWOASs) and phosphates in restored or unrestored soils and (2) to assess the potential importance of LMWOASs and phosphates for As mobilization with natural soil microbial communities in restored and unrestored soils.

## 2. Materials and Methods

### 2.1. Soil Sampling and Pretreatment

Soil samples were collected in July 2021 from the surface layer (0–20 cm) of As-contaminated soil in the transition zone between agricultural land and a river in the vicinity of a gold mine in Dandong, Liaoning Province, China (123°42′ E, 40°44′ N). After collection, the soils were air-dried and then passed through a 2 mm sieve. The characterization methods for each parameter are summarized in the Supplementary Materials [41,42].

Then, part of air-dried and sieved soil underwent sterilization (121 °C for 1 h) using steam sterilization pot GI54T (Zealway, Xiamen, China) to obtain the sterilized soil. Five percent (by weight) of nZVI was mixed thoroughly with 20 g of sterile soil in a 100 mL beaker (group of 40 samples, consisting of restored soil). Nano zero-valent iron (nZVI) was supplied by Xindun Co., Ltd. (Nangong, China). The remaining sterile soil samples (group of 40 samples, consisting of unrestored soil) without additions were used as a control. The mixture was homogenized with a water-holding capacity of ~70% at room temperature for 7 days. The subsamples were then freeze-dried, and the soil-available As was extracted with 0.5 M NaHCO_3_ [43].

### 2.2. Microcosm Experiments

Ten grams of original sieved soil was suspended in 50 mL of sterile 10 mM Tris-HCl buffer (pH 7.2). These soil suspensions were then used as a source of soil microbial communities.

#### 2.2.1. Experiment I (LMWOASs)

Two grams of dried soil subsample was weighed in 20 mL of brown serum vials and autoclaved at 121 °C for 1 h. Then, sterilized oxalate, citrate, malate, lactate, acetate, and phytate solutions (10 mL) were added separately to the sterilized restored or unrestored soil as a carbon source. Specifically, sodium oxalate, sodium citrate, sodium malate, sodium lactate, sodium acetate, and sodium phytate were added at concentrations of 11.17, 7.17, 7.42, 6.23, 6.84, and 25.66 g/L, respectively, to obtain an initial concentration of total organic carbon (TOC) of LMWOASs of 2 g/L. Sodium hydroxide and hydrochloric acid were used to regulate the pH of the LMWOAS solutions to 7.2. The incubation samples were N_2_-bubbled for 5 min. Each soil suspension (1 mL) was then inoculated into the serum vials in biotic groups, and the remaining soil suspensions were autoclaved (121 °C, 1 h) and then inoculated in abiotic controls. Finally, the samples were incubated under anaerobic conditions at 30 °C in the dark on a reciprocal shaker (120 rpm). All treatments were carried out in triplicate. The soil was sampled after 3, 10, 17, 24, and 38 days of incubation.

#### 2.2.2. Experiment II (Phosphates)

Microcosm incubations were constructed in 20 mL of brown serum vials containing 2 g of sterilized restored or unrestored soil and 10 mL of sterilized medium. The salt medium supplement used in this study was modified from Yamamura et al. (2003) and contained (per L): (NH_4_)_2_SO_4_ (2.27 mM), MgSO_4_·7 H_2_O (0.57 mM), NaCl (1.71 mM), KH_2_PO_4_ (0.0, 0.07, 0.35 mM), Na_2_HPO_4_·12 H_2_O (0.0, 0.06, 0.30 mM), C_3_H_5_O_3_Na (20 mM), and 1 mL of trace element solution [44]. The final concentrations of P in the medium were 0 mM, 0.13 mM, and 0.65 mM. The pH of the medium was adjusted to 7.2. The incubation samples were N_2_-bubbled for 5 min, and each soil suspension (1 mL) was then inoculated into the serum vials. Finally, the samples were incubated under anaerobic conditions at 30 °C in the dark on a reciprocal shaker (120 rpm). All treatments were carried out in triplicate. The soil was sampled after 1, 2, 3, 4, 5, 7, 9, and 11 days of incubation.

### 2.3. Geochemical Analysis

The pH and oxidation–reduction potential (ORP) of the microcosm slurries were measured using a PB-10 device (Sartorius, Germany) and a portable ORP meter (HACH, Shanghai, China), immediately after sampling. When HCl-extractable Fe(II) was analyzed, 1 mL of 2 M HCl was mixed with the same volume of slurry and then mixed vigorously before filtration [45]. The supernatants in the brown serum vials were collected and filtered through a 0.22 μm polytetrafluoroethylene (PTFE) membrane after centrifugation (1100× *g* for 10 min) with a 3K15 high-speed centrifuge (SIGMA, Germany). Slurries were also filtered through a 0.22 μm filter. Fe(II) was quantified using the 1,10-phenanthroline colorimetric method using a UV1800 spectrophotometer (Shimadzu, Japan) [46], which was conducted immediately. Part of the filtrate was acidified with 0.1 M HNO_3_, and then total As, Fe, and P were analyzed by inductively coupled plasma–optical emission spectroscopy (ICP–OES) (Spectro Blue Sop, Germany). The ferric iron was calculated from the concentrations of total iron and ferrous iron. TOC levels were determined using TOC-L CPH equipment (Shimadzu, Japan). The instrument parameters for ICP−OES and TOC-L CPH equipment are summarized in the Supplementary Materials [41,42].

### 2.4. Quality Assurance/Quality Control (QA/QC) and Statistical Analysis

For quality assurance and quality control, measures for quality assurance and quality control were taken in the experimental design and laboratory analyses. All control and experimental treatments were carried out in triplicate throughout the whole experiment. In routine analysis, for every 10 samples, 1 laboratory blank, 1 parallel sample, and 1 spiked blank sample were added to the measuring sequence. If the sample number was less than 10, then 1 laboratory blank, 1 parallel sample, and 1 spiked blank sample were also taken.

In our analysis, the concentrations of target substances in all blanks were less than the corresponding MDLs. The standard solutions were provided by Beijing Wanjia Shouhua Biotechnology Co. (Beijing, China). The Chinese national standard was provided by the National Research Center for certified Reference Materials of China. The recovery range of spiked blank was 94–103%. The relative standard deviation (RSD) was lower than 5% for all of the tests. All statistical analyses were performed using IBM SPSS Statistics version 22.0.

## 3. Results and Discussion

### 3.1. Arsenic Release under LMWOAS Treatments in Unrestored Soil

The basic physical and chemical properties of the soils are given in Table 1. The results of other characterizations are shown in the Supplementary Materials [41,42].

**Figure 1:**
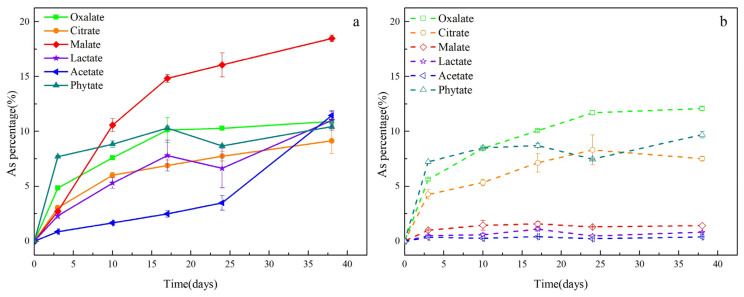
Dissolved As release as a percent of total As in soil under biotic (**a**) and abiotic (**b**) conditions with different LMWOAS amendments in unremediated soils.

A comparative analysis under different LMWOAS treatments revealed significant differences (*p* < 0.05) in As release from in situ soil (Figure 1). The effectiveness of As release under the LMWOAS treatments in abiotic control assays are ordered as follows: oxalate > phytate > citrate > malate > lactate > acetate. However, for the biotic microcosms, the dissolved As released from each group changed over time. The final rank order of the treatments after 38 days was as follows: malate > acetate ≈ lactate ≈ oxalate ≈ phytate > citrate. The maximum release of As in the biotic control assay was observed with the addition of malate, which released 18.47% of the arsenic in the soil.

**Figure 2:**
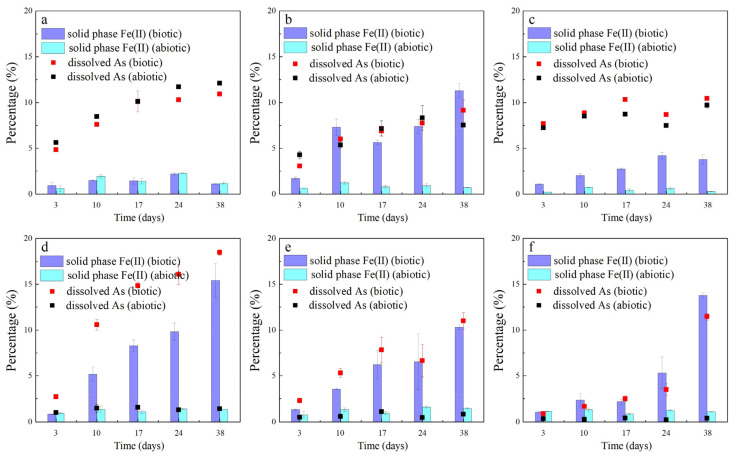
Variation in dissolved As in the supernatant and solid-phase Fe(II) as percent extracted of total in soil under biotic and abiotic conditions with different LMWOAS amendments in unremediated soils. (**a**) Oxalate, (**b**) citrate, (**c**) phytate, (**d**) malate, (**e**) lactate, (**f**) acetate.

Biotic and abiotic control groups treated with the same LMWOAS were then further analyzed. In all abiotic controls, minor As release occurred in the malate, lactate, and acetate treatment groups. However, in the oxalate, citrate, and phytate treatments, significant amounts of As were still released under sterilized conditions, and there were small differences from the unsterilized conditions. Compared to the abiotic control assays, the microorganisms inhibited the release of As in oxalate treatments, and the release of As in the other five treatments was promoted (Figure 2). In all biotic cases, As release from the slurries failed to plateau during the experimental period, suggesting the potential for further release of As with longer incubation times.

**Figure 3:**
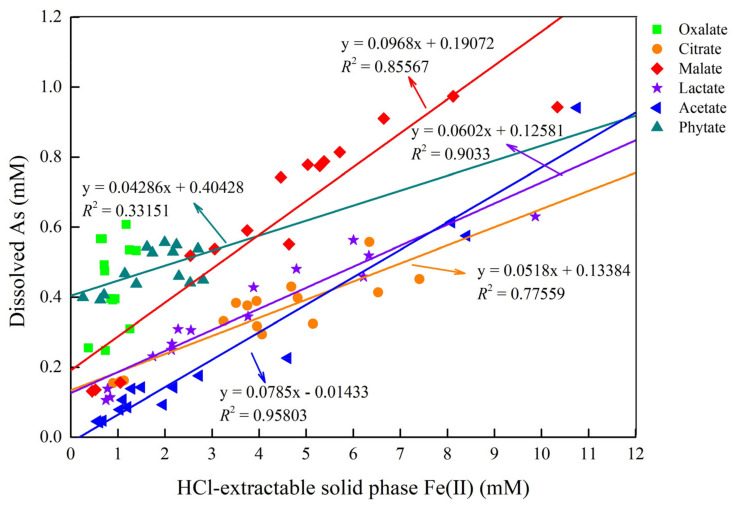
Relationships between dissolved As and HCl-extractable solid-phase Fe(II) for unremediated soil with different LMWOAS amendments.

Low-molecular-weight dissolved organic carbon can enhance the biotic or abiotic reductive dissolution of iron oxides, oxyhydroxides, and hydroxides in the presence or absence of Fe(III)-reducing microbial communities [47]. In addition, HCl-extractable Fe(II) is biologically available to microorganisms, and it could represent predominant biological and chemical sources of Fe(II) species produced by ferric iron reduction and could keep their concentration and valence state stable during the extraction [48]. The HCl-extractable total Fe(II) includes dissolved and solid-phase Fe(II). Since the Fe(II) content of the supernatant constituted a very small proportion of the HCl-extractable total Fe(II), no significant correlation was found between As and Fe(II) in the aqueous solution (Figure S1). Therefore, only the solid-phase Fe(II) concentration was further analyzed. Data for solid-phase Fe(II) for the six samples are shown in Figure 2. In general (except for the oxalate treatment), the solid-phase Fe(II) concentrations of the remaining five biotic control groups were much higher than those of the abiotic control groups. In contrast, there was no clear relationship between the solid-phase Fe(II) or soluble As in different LMWOAS treatments. However, when the data from the six soil samples are plotted together, except for the oxalate treatment, which showed no relationship, a significant relationship between the solid-phase Fe(II) and soluble As for the other individual soil samples was observed (*p* < 0.05). The *R*^2^ value for the five soil samples ranged from 0.332 to 0.958 (Figure 3).

Song et al. (2010) showed that the primary mechanism by which low-molecular-weight organic acids or their salts promote the release of heavy metals may not reside in the action of acid but rather in the action of organic anions [49]. The solid-phase Fe(II) concentrations of the malate, lactate, and acetate treatments in the abiotic control group always showed very little variation, and the release of As was always low. This result indicates that these three LMWOASs have a low capability of reductive or non-reductive dissolution of iron minerals in soils under sterile conditions. The competitive desorption ability of As was weak under sterile conditions. However, with the application of these three LMWOASs, the participation of microorganisms significantly promoted the reductive dissolution of iron minerals and greatly enhanced the release of As from the soil. The changes in As and solid-phase Fe(II) concentrations in lactate and acetate treatment groups showed a similar tendency to that observed by Wang et al. (2021) [50]. One possible reason that the largest amount of iron reduction and arsenic release was observed in the biotic malate treatments is the mitochondrial Krebs cycle in fungi [51]. Several experiments have demonstrated that malate is closely associated with the migration and transformation of Fe and As in the plant rhizosphere [52,53]. The results show that microorganisms probably affect the behavior of As in the LMWOA–transition zone soil mixtures.

The citrate and phytate treatment groups released large quantities of As under sterile conditions. Additionally, the Fe(II) concentration in the sterile supernatant was also consistently high (Figure S2). Both the solid-phase Fe(II) concentration and As release increased after microbial involvement in the reaction. This indicates that, on the one hand, the hydroxyl group and the carboxyl group of citrate and the orthophosphate moieties of phytate can promote the abiotic release of As and Fe(II) from soils. On the other hand, microbial action can develop a synergy with abiotic action to further promote As release from the LMWOAS–transition zone soil mixtures.

The oxalate treatments showed little difference in the solid-phase Fe(II) concentration in the presence or absence of microorganisms. The differences in Fe(II) concentration suggest that the chemistry of oxalate is mainly responsible for promoting the reaction with iron minerals. The release rate of As gradually decreased in oxalate treatments. The insoluble Fe(III)–organic complexes that formed covered the surface of the binding site and led to a block in the release of ions [54]. Since the release of arsenic is mainly the result of the chemical role of oxalate, the phenomenon becomes more pronounced in oxalate treatments. Additionally, based on the release of As from the solution, microorganisms inhibit the release of As from the soil in the presence of oxalate. This result is consistent with that of Mei et al. (2022), who found that microorganisms may facilitate the release of As from sediments in the presence of citric and malic acids, but they suppress As mobilization in the presence of oxalic acid. After sterilization, the As extraction from sediments by citric and malic acids decreased, whereas the extraction by oxalic acid increased [39].

### 3.2. Arsenic Release under LMWOAS Treatments in Restored Soil

**Figure 4:**
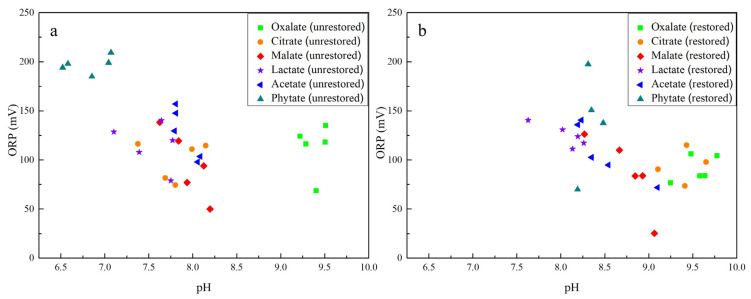
Soil pH and ORP distribution under biotic conditions with different LMWOAS amendments in unrestored (**a**) and restored (**b**) soils.

The remediation effect was determined by NaHCO_3_ extraction after 7 days’ remediation by applying excess nZVI. According to the available As concentration extracted before and after the remediation, the repair efficiency was ~70%. Compared with the unrestored soil, there was little change in the ORP range throughout the experiment. However, the pH of the soil slurry increased in restored soil due to the oxidation of nZVI and the release of OH- (Figure 4) [55]. The citrate and phytate treatments showed a greater change (pH and ORP in sterilized incubations are not shown).

**Figure 5:**
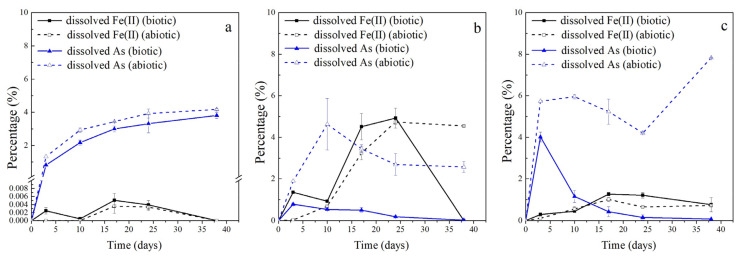
Variation in dissolved As and Fe(II) in the supernatant as percent extracted of total in soil under biotic and abiotic conditions with different LMWOAS amendments in remediated soils. (**a**) Oxalate, (**b**) citrate, (**c**) phytate.

For amendments with different LMWOASs, the concentrations of dissolved As and Fe(II) in the supernatant are shown in Figure 5. Only Fe(II) in the supernatant was analyzed due to the problem of significant interference with HCl-extractable Fe(II) with the application of excessive nZVI.

In the restored soil, with the application of excess nZVI, the soluble Fe(II) in the malate, lactate, and acetate amendments showed similar trends compared to the unrestored soil (Figure S3). Relatively minor As release occurred, regardless of the presence or absence of microorganisms in the soil. According to these data, we speculate that excess nZVI led to a significant increase in the number of adsorption sites. Hence, the As released by either microbial reductive dissolution or desorption was immediately readsorbed.

Although the release of As initially occurred with oxalate, citrate, and phytate amendments in restored soil, the release was significantly lower than that in unrestored soil. On the one hand, nZVI reduced As mobility. On the other hand, oxalate, citrate, and phytate could maintain As release. The added oxalate extensively formed strong complexes with iron, which can prevent the precipitation of a new iron oxide phase and can inhibit the mechanisms of nZVI repair during As release [56]. Under near-neutral pH conditions, Fe(III)–citrate can accelerate As release in the presence of arsenopyrite [57]. It was reported that phytic acid solubilized 39% of poorly soluble FeAsO_4_, while citric and oxalic acid solubilized 32 and 10% because of their stronger complex stability with Fe [58]. Moreover, the As(V) re-release amount changed with the aging time of ferrihydrite. The effect of organic ions such as oxalate, citrate, and phytate on the As(V) release tendency is associated with factors such as the As loading rate and ligand concentration in Fe–As complexes and leads to higher As(V) re-release after longer ferrihydrite aging time [59].

At the beginning of the experiment, the magnitude of As release in the abiotic control group occurred in the following order: phytate > oxalate ≈ citrate amendment. In the biotic control groups, the release of As occurred in the following order: phytate > oxalate > citrate amendment. In the oxalate treatment groups, the trend of the release of As and Fe(II) in the restored soil was the same as that in the unrestored soil. The As release increased gradually over time, but microorganisms inhibited its release.

Due to the citrate and phytate and microorganisms acting synergistically, significant dissolution release of Fe(II) occurred in the restored soil with the addition of citrate and phytate. With the participation of microorganisms, the release of As showed an initial increasing trend, followed by a decreasing trend. The reason for this may be because As was initially released by synergistic action. However, because of large amounts of soluble Fe(II) in the solution and the alkaline soil pH, secondary minerals were formed that captured As, leading to its re-immobilization. A similar phenomenon was observed by Wang et al. (2021) and Cai et al. (2020) [50,60]. Compared to the experimental biotic group, the amount of As released was much greater, although the abiotic control group also released significant amounts of Fe(II). In parallel, the sterilized and unsterilized groups had similar pH values (Figure S4). Therefore, we speculate that microorganisms actively participate in the formation of secondary minerals under such conditions.

### 3.3. Effect of Phosphate on as Release in Restored and Unrestored Soils

**Figure 6:**
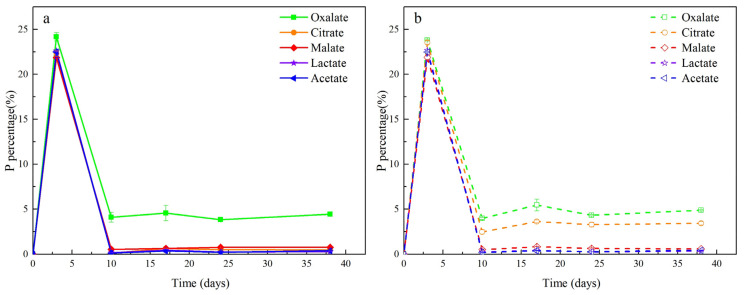
Dissolved P release as a percent of total P in soil under (**a**) biotic and (**b**) abiotic conditions with different LMWOAS amendments in unremediated soils.

During previous experiments with LMWOASs, the concentration of P in the supernatant was also measured. Because phytate itself carries a large quantity of P, which interferes with the measurement results, the phytate treatment group was excluded when comparing P concentrations. The remaining LMWOAS groups in the abiotic and biotic groups had similar P concentrations (Figure 6). The maximum P concentration occurred on the 3rd day, and subsequently, on the 10th day, the concentrations decreased and then remained stable. This phenomenon suggests that the release of P is due to the perturbation of the soil environment caused by the addition of the solution. By the third day, there was a significant correlation between the As and P concentrations in the supernatant of the five groups (*p* < 0.01). This likely demonstrates the physicochemical effect of P on the release of As in the initial period of the reaction. Therefore, we investigated whether the role of exogenous P input on As release was facilitated by the participation of microorganisms.

**Figure 7:**
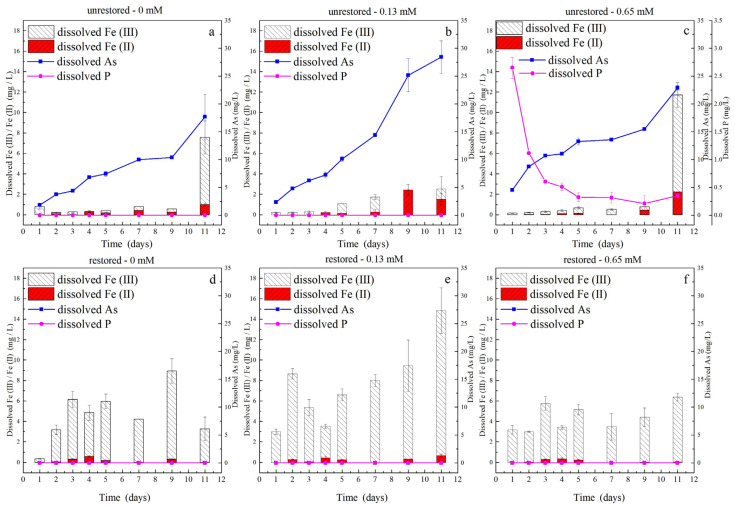
Variation in dissolved As, Fe(II), and Fe(III) concentrations in the supernatant under biotic conditions with different phosphate concentrations in unrestored and restored soils. (**a**) Unrestored, 0 mM, (**b**) unrestored, 0.13 mM, (**c**) unrestored, 0.65 mM, (**d**) restored, 0 mM, (**e**) restored, 0.13 mM, (**f**) restored, 0.65 mM.

The changes in soluble P and As concentration in the unrestored soil are shown in Figure 7. The results clearly show that high P concentrations increase the initial As release, which was evident in the results for the 1st day. The higher the P concentration applied, the greater the As release, which is probably because phosphate promotes the release of As from the soil via competitive adsorption. However, it should be noted that the release of As still increased when the P concentration leveled off. This may be because the presence of phosphate promotes microbial action, resulting in persistent As release. Arsenate and phosphate create competition for the same transport channel protein. Phosphate stimulated As release due to fast bacterial reproduction because increasing phosphate concentration in the environment appears to decrease the growth inhibition attributed to the presence of arsenate [32].

**Figure 8:**
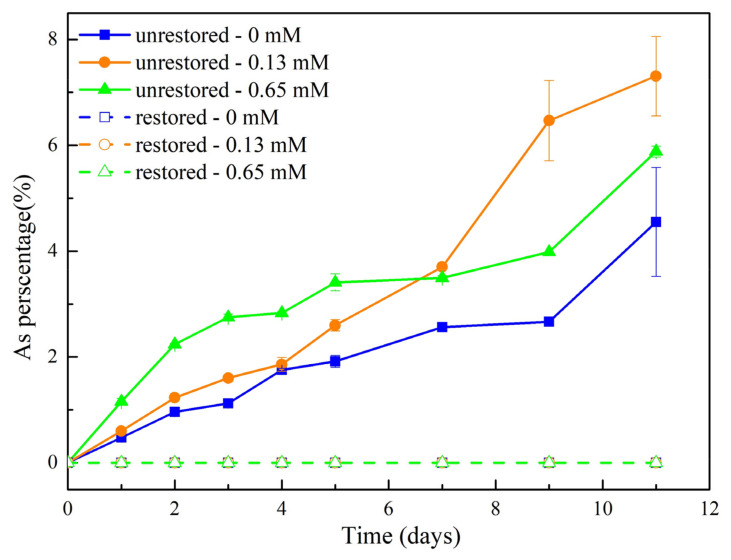
Dissolved As release as a percent of total As in soil under biotic conditions in restored and unrestored soils with different phosphate concentrations.

**Figure 9:**
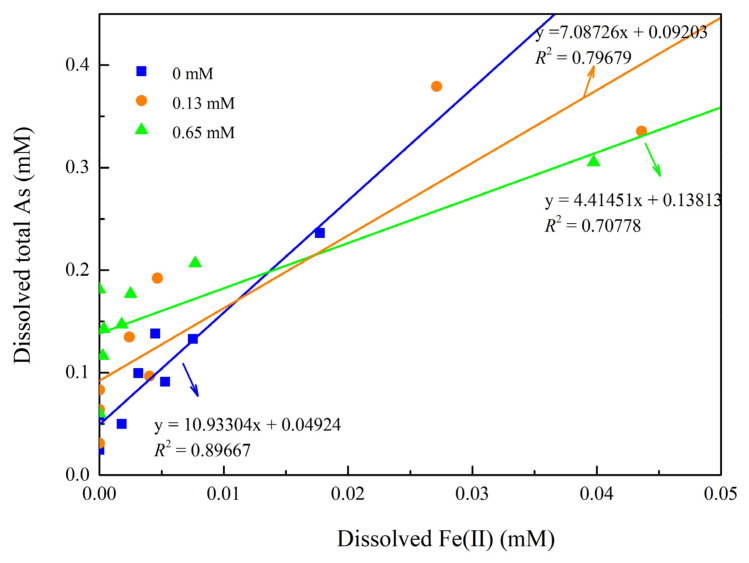
Relationship between dissolved As and Fe(II) for unremediated soil with different phosphate concentrations.

Although we found a higher initial release of As in the 0.65 mM phosphate groups, the 0.13 mM phosphate groups eventually released the most As at the end of the reaction (Figure 8). The proportion of soluble Fe(II) in the released dissolved Fe was the highest in the 0.13 mM phosphate groups. One possible explanation for the lower Fe(II) levels in the 0.65 mM phosphate groups is that dissolved Fe(II) reacts with the excess P to precipitate as Fe_3_(PO_4_)_2_ (vivianite). A similar phenomenon was observed by Zhang et al., (2017); additionally, the lack of P may lead to lower microbial activity in the 0 mM phosphate groups [14]. A significant positive correlation (*p* < 0.01) was observed between soluble Fe(II) and As in the solutions of the three groups (Figure 9).

In the unrestored soil, the P concentration in the 0.65 mM phosphate groups gradually decreased and eventually remained stable, but we did not detect the release of P in the supernatant in the 0 and 0.13 mM phosphate groups. It was previously suggested that As is translocated with Fe during redox changes, whereas phosphate is not affected by redox, likely because phosphate is adsorbed onto non-reduced iron oxides [61]. Therefore, it is possible that we could not observe P in the supernatant because the original P in the soil and the low concentration of exogenous P were stabilized in the soil by the soil matrix. While the soil could not completely absorb the high concentration of exogenous P input at the beginning, it was gradually stabilized in the soil as the reaction progressed. In the restored soil, even high concentrations of exogenous P were immobilized by the presence of excess nZVI, so no P release was observed in the supernatant.

No As release was observed in the restored soil even when more iron was released (Figure 7). In particular, in the 0.65 mM phosphate groups, neither As nor P was released into the supernatant. We speculate that the phenomenon is due to the high affinity of the Fe oxide shell of nZVI for PO_4_^3−^ through surface complexes including electrostatic attraction and chemical binding (Fe-O-P) [62]. The result showed a similar tendency to that observed by Huang et al. (2019) [63]. The experimental results show that excess nZVI may, to some extent, avoid the competitive desorption caused by phosphate input and the effect of the Fe reduction process promoted by microorganisms.

## 4. Conclusions

The secondary release of As in rhizospheric soil has been neglected in previous studies and deserves special attention. Microorganisms play the dominant role in the process of As release, and the interactions of As, microorganisms, LMWOASs, and phosphates during the process were investigated in this study. Microorganisms showed complex inhibition or facilitation effects during the release of As. NZVI is widely applied for the remediation of As-contaminated soil, and it suppressed the majority of As release in this study. This study improves our understanding of As mobilization and transformation in the rhizosphere and helps to provide an effective strategy for reducing the secondary release of As from soils due to replanting after restoration.

## Figures and Tables

**Table 1:** Basic physical and chemical properties of the studied soil.

Property	Value/Content
pH	7.61
Organic matter/(mg/kg)	10,500
Total C/(mg/kg)	14,500
Total P/(mg/kg)	549
Total S/(mg/kg)	3200
Total N/(mg/kg)	636
Total Fe/(mg/kg)	33,400
Total As/(mg/kg)	1944
Available As/(mg/kg)	35

## Data Availability

The data that support the findings of this study are available from the corresponding author, X. Luo (luoxm@cugb.edu.cn), upon reasonable request.

## Supplementary Materials

The following supporting information can be downloaded at: https://www.mdpi.com/article/10.3390/ijerph19084512/s1, Figure S1: Relationships between dissolved As and the dissolved Fe(II) for unremediated soil with different LMWOAS amendments; Figure S2. Dissolved Fe(II) release as a percent of total Fe in soil for unremediated soil with different LMWOAS amendments.(a) oxalate, (b) citrate, (c) phytate, (d) malate, (e) lactate, (f) acetate; Figure S3: Variation of dissolved As and Fe(II) in the supernatant as percent extracted of total in soil under biotic and abiotic conditions with different LMWOAS amendments in remediated soils.(a) malate, (b) lactate, (c) acetate; Figure S4: Soil pH under biotic and abiotic conditions with citrate and phytate amendments in restored soils; Table S1: XRD results of phreatic aquifer soil sample; Table S2: Analysis results of phreatic aquifer soil sample; Table S3: Arsenic fraction percentage in the different fractions of experimental soil; Table S4: Reagent Manufacturers.
