# Supplementary material for: Arsenic Release from Soil Induced by Microorganisms and Environmental Factors
Source: Int J Environ Res Public Health. 2022 Apr 8;19(8):4512. doi: 10.3390/ijerph19084512 (PMC9027750; doi:10.3390/ijerph19084512)
Supplement: Supplementary file 1 [file ijerph-19-04512-s001.zip › ijerph-1633920-supplementary.pdf]

# Supplementary Materials

# Arsenic release from soil induced by microorganisms and environmental factors

**Yitong Yin [1], Ximing Luo [1,2*], Xiangyu Guan[1,2], Jiawei Zhao[1], Yuan Tan[1], Xiaonan Shi[1], Mingtao Luo[1] and Xiangcai Han[1,3]**

[1] School of Ocean Sciences, China University of Geosciences (Beijing), Beijing, 100083, China ; kaqichuan@163.com(Y.Y.)

[2] Beijing Key Laboratory of Water Resources and Environmental Engineering, China University of Geosciences (Beijing), Beijing, 100083, China; luoxm@cugb.edu.cn(X.L.)

[3] Yantai Coastal Zone Geological Survey,China Geological Survey, Yantai,China

* Correspondence: luoxm@cugb.edu.cn

AUTHOR INFORMATION

Corresponding Author

*Phone: +86 138 0105 0133; e-mail: luoxm@cugb.edu.cn

Number of pages (including cover sheet): 6

Number of tables: 4

Number of figures: 4

*Characterization of the soils*

The samples used to analyze the physicochemical properties and metal elements were air-dried at room temperature and then passed through sieve. pH was measured in a 1:2.5 (soil:water) mixture using a PB-10 device (Sartorius, Germany). Soil total organic matter (OM) content was measured by the $K_2Cr_2O_7$-$H_2SO_4$ wet oxidation method. The total nitrogen (TN) was measured following the Kjeldahl method. The carbon (C) and sulfur (S) contents of the soil were analyzed using a high-frequency infrared carbon and sulfur analyzer BCS-801A(Science, Sichuan, China). The phosphorus (P) and iron (Fe) contents of the soil were determined using a X-ray fluorescence spectrometer ZSXPrimusII (Rigaku, Japan). The total arsenic(As) in the soil was analyzed by atomic fluorescence spectrometry (AFS)(Haiguang, Beijing, China). Available As in the soil was extracted using 0.5 M $NaHCO_3$, with the soil to solution ratio of 1:10 and then analyzed by inductively coupled plasma–optical emission spectroscopy (ICP−OES)(Spectro Blue Sop, Germany).

*Instrument parameters*

The filtrates for analyzing total As, Fe and P were acidified with 0.1 M $HNO_3$ before testing on the machine. The parameters of inductively coupled plasma–optical emission spectroscopy (ICP−OES)(Spectro Blue Sop, Germany) were set as: Helium (99.999%) was used as carrier gas, the plasma power was kept at 1300W, cooling gas at a flow rate of 14 L/min, auxiliary gas at a flow rate of 1 L/min, nebulizing gas at a flow rate of 0.8 L/min.

The TOC content in the corresponding filtrates was measured using TOC-L CPH equipment(Shimadzu, Japan). Instrument parameters were as follows: mixed gas with a mixture of 21% $O_2$ and 79% $N_2$ was used as carrier gas, the flow rate was 150 mL/min, the pressure set to 200 kPa. The electric oven temperature was held 720 ℃. The reaction solution was 25% phosphoric acid.

*Properties of the soil sample*

The mineral phases, main compositions, and the arsenic existing form of the mine tailings near the sampling site have been already analyzed by X-ray Diffraction, X-ray Fluorescent and Electro-probe Microanalysis, respectively. The main mineral compositions of flotation mine tailing are quartz, dolomite and clay minerals and their chemical components are mainly Si and Al. The color of mine tailing is off-white. The arsenic content of this mine tailing is about 754 μg/g, mainly existing in the form of arsenopyrite ( FeAsS)[1].

Table S1. XRD results of phreatic aquifer soil sample[2].

| Mineral species and content(%) | | | | | | |
|---|---|---|---|---|---|---|
| quartz | potassium feldspar | plagioclase | dolomite | hornblende | gypsum | clay minerals |
| 54.3 | 6.4 | 6.9 | / | 2.1 | / | 30.3 |

Table S2. Analysis results of phreatic aquifer soil sample[2].

| Clay mineral species and content(%) | | | | | | mixed layer ratio(%) | |
|---|---|---|---|---|---|---|---|
| Smectite | Illite | Kaolinite | Chlorite | Illite/Smectite mixed layer | Chlorite/Smectite mixed layer | Illite/Smectite mixed layer | Chlorite/Smectite mixed layer |
| / | 34 | 5 | 9 | 44 | 8 | 30 | 45 |

Table S3. Arsenic fraction percentage in the different fractions of experimental soil.

| As fraction (%) | | | | | | |
|---|---|---|---|---|---|---|
| Water-soluble fraction | Exchangeable fraction | Carbonate bound fraction | Humic acid bound fraction | Fe/Mn oxide bound fraction | Organic matter bound fraction | Residual fraction |
| 0.88 | 0.48 | 4.64 | 4.37 | 2.32 | 2.05 | 85.26 |

Table S4. Reagent Manufacturers.

| Reagent | Manufacturers |
|---|---|
| Tris-HCl (1M) | Schleden |
| $C_4H_4Na_2O_5$ (>98.0%(T)) | Aladdin |
| $C_2O_4Na_2$(AR) | Aladdin |
| $CH_3COONa$(AR) | Aladdin |
| $C_6H_5Na_3O_7$(AR) | Aladdin |
| $C_3H_5O_3Na$(AR) | Aladdin |
| $C_6H_6O_{24}P_6$.Na(>90%(dry basis)) | Aladdin |
| $NaHCO_3$(AR) | Sinopharm Chemical Reagent Co., Ltd |
| $HNO_3$(68%) | Beijing Chemical Works |
| $H_3PO_4$(85%) | Aladdin |
| $H_2SO_4$(98%) | Beijing Chemical Works |
| HCl(37%) | Beijing Chemical Works |
| NaOH(AR) | Sinopharm Chemical Reagent Co., Ltd |
| $(NH_4)_2SO_4$(AR) | Sinopharm Chemical Reagent Co., Ltd |
| $MgSO_4·7\ H_2O$(AR) | Sinopharm Chemical Reagent Co., Ltd |
| NaCl (AR) | Sinopharm Chemical Reagent Co., Ltd |
| $KH_2PO_4$(AR) | Sinopharm Chemical Reagent Co., Ltd |
| $Na_2HPO_4·12\ H_2O$(AR) | Sinopharm Chemical Reagent Co., Ltd |
| 1,10-phenanthroline(AR) | Tianjin Guangfu Chemical Research Institute |
| $K_2Cr_2O_7$(AR) | Sinopharm Chemical Reagent Co., Ltd |

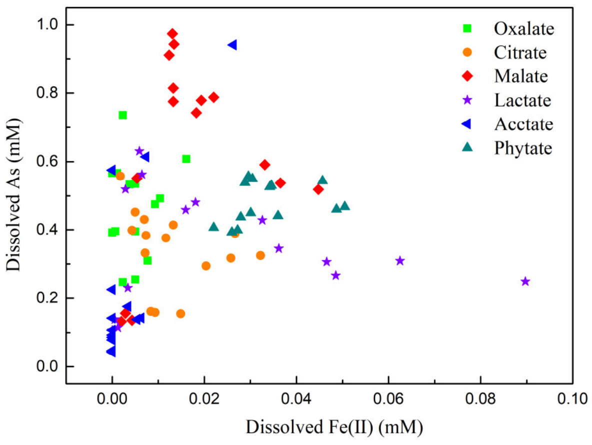


Figure S1. Relationships between dissolved As and the dissolved Fe(II) for unremediated soil with different LMWOAS amendments.

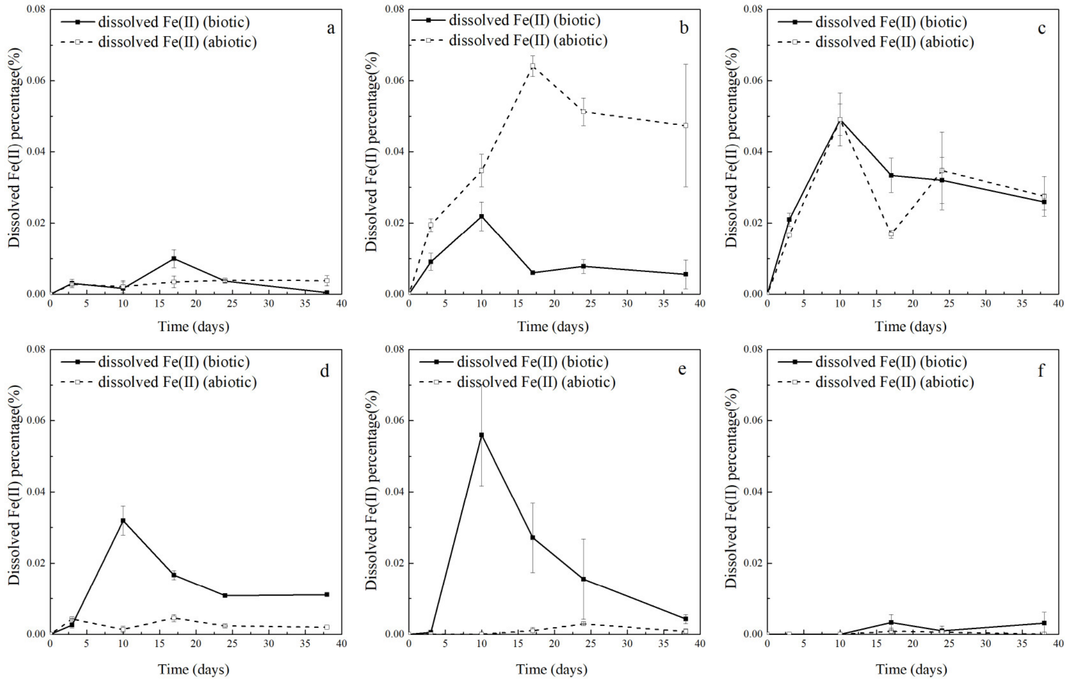


Figure S2. Dissolved Fe(II) release as a percent of total Fe in soil for unremediated soil with different LMWOAS amendments.

(a) oxalate, (b) citrate, (c) phytate, (d) malate, (e) lactate, (f) acetate.

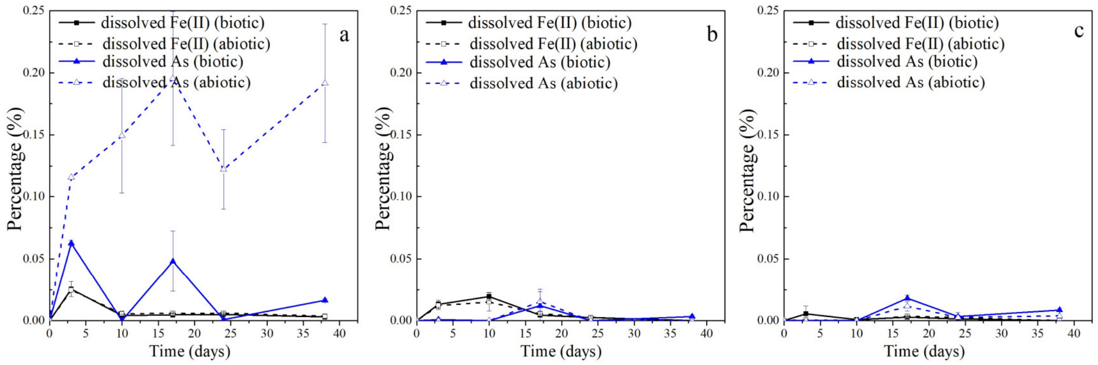


Figure S3. Variation of dissolved As and Fe(II) in the supernatant as percent extracted of total in soil under biotic and abiotic conditions with different LMWOAS amendments in remediated soils.(a) malate, (b) lactate, (c) acetate.

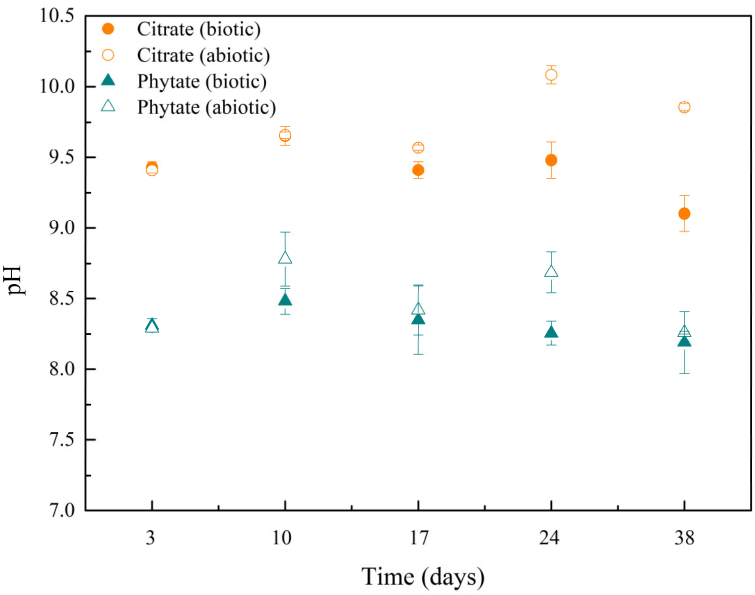


Figure S4. Soil pH under biotic and abiotic conditions with citrate and phytate amendments in restored soils.

References
1. Z. Chen, D. Li, X. Luo, Research on arsenic form in the gold mine tailings by different leaching processes. *Rock and Mineral Analysis*, **2014**,*33*,363-368.
2. X.M Luo, Case study on the migration and transform of arsenic in aquatic environment caused by gold mining — Dandong, Liaoning. Doctoral Degree, China University of Geosciences(Beijing), Beijing, 2007.
